# Understanding the preterm human heart: What do we know so far?

**DOI:** 10.1002/ar.24875

**Published:** 2022-02-09

**Authors:** Art Schuermans, Adam J. Lewandowski

**Affiliations:** ^1^ Oxford Cardiovascular Clinical Research Facility, Division of Cardiovascular Medicine, Radcliffe Department of Medicine University of Oxford Oxford UK; ^2^ Faculty of Medicine KU Leuven Leuven Belgium

**Keywords:** cardiac remodeling, cardiovascular risk, prematurity, preterm heart, transitional physiology

## Abstract

Globally, preterm birth affects more than one in every 10 live births. Although the short‐term cardiopulmonary complications of prematurity are well known, long‐term health effects are only now becoming apparent. Indeed, preterm birth has been associated with elevated cardiovascular morbidity and mortality in adulthood. Experimental animal models and observational human studies point toward changes in heart morphology and function from birth to adulthood in people born preterm that may contribute to known long‐term risks. Moreover, recent data support the notion of a heterogeneous cardiac phenotype of prematurity, which is likely driven by various maternal, early, and late life factors. This review aims to describe the early fetal‐to‐neonatal transition in preterm birth, the different structural and functional changes of the preterm human heart across developmental stages, as well as potential factors contributing to the cardiac phenotype of prematurity.

## 1 INTRODUCTION

Preterm birth, defined as delivery before 37 completed weeks of gestation, has a substantial impact on global healthcare. Over 15 million neonates are born prematurely each year, which corresponds to more than 10% of live births worldwide (Blencowe et al., 2012). Preterm birth can be further subdivided by gestational age into moderate‐to‐late preterm (32 to <37 weeks), very preterm (28 to <32 weeks), and extremely preterm (<28 weeks) birth, respectively, accounting for 85, 10, and 5% of all preterm births (Blencowe et al., 2012).

It is widely known that preterm birth associates with various morbidities such as necrotizing enterocolitis, sepsis, patent ductus arteriosus, visual and hearing problems, and neurological and respiratory diseases (Benitz *et al*., 2016; Platt, 2014; Vogel et al., 2018), and that it remains the leading cause of under‐five mortality globally (Liu et al., 2016). However, advances across the continuum of birth care, including maternal care, delivery techniques, and management of neonatal diseases, have made it possible for more prematurely born infants that are smaller and born earlier to reach adulthood. As these improvements in perinatal care are now widely established into clinical practice and increasingly more data about prematurely born adults become available, the long‐term cardiovascular consequences of preterm birth are becoming apparent. Notably, large‐scale epidemiological studies have shown that prematurely born adults have a higher chance of developing new‐onset heart failure (Carr et al., 2017; Crump et al., 2021) and ischemic heart disease (Crump et al., 2019; Ueda et al., 2014), among other cardiovascular diseases (Lewandowski et al., 2020), including early cardiovascular‐related mortality (Risnes et al., 2021).

Since prematurely born adults have a higher risk of developing cardiac disease, researchers have been investigating whether they exhibit distinct changes in heart structure and function that may increase their disease risk. Despite the studies describing functional differences in the neonatal preterm heart dating back to 1986 (Walther, Siassi, King, & Wu, 1986; Walther, Siassi, & Wu, 1986), the first comprehensive study investigating cardiac geometry and function in preterm‐born adults was not published until 2013 (Lewandowski, Augustine, et al., 2013). Numerous observational studies since then have explored the preterm heart through various developmental stages from birth to adulthood, using echocardiography and cardiovascular magnetic resonance (CMR) imaging. This review aims to give insight into the different structural and functional alterations of the premature heart, the potential driving forces of its formation, and the possible implications for disease risk and development.

## 2 THE FETAL‐TO‐NEONATAL TRANSITION AND THE PRETERM HEART

With preterm birth, the transition from an intrauterine to extrauterine environment occurs during a critical phase of development. Normally, this transition involves an initial strong and then later more gradual decrease in pulmonary vascular resistance following lung inflation, causing pulmonary blood flow and left atrial pressure to rise rapidly (Wu et al., 2016). Around the same time as the neonate takes its first breaths, the umbilical cord is cut, causing a sudden increase in systemic vascular resistance (Finnemore & Groves, 2015). Rather than being able to overcome these circulatory changes, the immature cardiovascular system of the premature newborn becomes highly susceptible to them. This is because their immature heart has low inherent contractility, poor tolerance of high systemic vascular resistance, impaired diastolic function, and persistent fetal shunt pathways, all of which impede a normal fetal‐to‐neonatal transition (Bensley et al., 2016; Finnemore & Groves, 2015).

Animal models and histomorphological studies have shown that the premature interruption of cardiac growth leads to cellular changes in the myocardium. In different reports on preterm‐born sheep, the myocardium was characterized by hypertrophied cardiomyocytes in both ventricles (Bensley et al., 2010), alterations in the extracellular compartment (Le, Ferreira, et al., 2021), and an increase in interstitial collagen deposition (Bensley et al., 2010; Le, Dahl, et al., 2021). Similarly, in a rat model of neonatal hyperoxia exposure to mimic preterm birth‐related conditions, the cardiomyocytes are hypertrophied and there is greater interstitial fibrosis, with in vivo signs of left ventricular (LV) dysfunction by echocardiography (Bertagnolli et al., 2014). In the only human cardiac autopsy study to date, Bensley et al. (2018) investigated myocardial tissue samples from neonates who died after preterm birth and compared them to samples from age‐matched control stillborn fetuses. The preterm myocardial tissue showed a marked reduction in cardiomyocyte proliferation compared to the fetal control samples, providing further evidence of a disruption in normal cardiomyocyte hyperplastic growth (Tan & Lewandowski, 2020).

## 3 CARDIAC PHENOTYPE(S) OF PREMATURITY

### 3.1 The preterm cardiac phenotype in the postnatal period

Studies in humans have provided insight into the effects of premature birth on cardiac structure and function in neonates. In terms of structure, studies have shown that individuals born preterm have a lower LV mass index than their term‐born peers at birth (Aye et al., 2017; Telles et al., 2020). Additionally, the preterm neonatal heart has a more globular shape and more spherical blood pool (Aye et al., 2017; Cox et al., 2019). However, during the first postnatal months, the preterm heart undergoes significant remodeling through an excessive increase in LV and right ventricular (RV) mass (Aye et al., 2017; Kozak‐Barany et al., 2001). Using CMR, Cox et al. (2019) found that preterm neonates at term‐corrected age had significantly greater weight‐indexed LV masses than term‐born neonates at birth. They also trended toward having greater weight‐indexed RV masses. The weight‐indexed LV masses were associated with the degree of prematurity, as infants born at <29 weeks of gestation showed a more than 60% increase in weight‐indexed LV mass at term‐corrected age than the term‐born control cohort. The authors also found that preterm‐born individuals had a higher weight‐indexed LV end‐diastolic volume at term‐corrected age. This was not seen in the follow‐up measurements using echocardiography in preterm infants at 3 months by Aye et al. (2017), but may reflect an enhanced physiological adaptation to the increased pulmonary venous return in neonates born at earlier gestations (Lewandowski, 2019).

Cardiac differences between preterm and term neonates are not limited to structure. A recent meta‐analysis comparing preterm‐ and term‐born neonates revealed that preterm newborns possess marked functional cardiac impairments (Telles et al., 2020). All analyzed measures of LV and RV systolic function were lower in preterm versus term neonates, including LV ejection fraction (EF), which showed a weighted mean difference of −2.89%. LV and RV longitudinal systolic strain were inferior as well, with weighted mean differences of 2.53 and 2.94%, respectively. Preterm neonates also showed biventricular diastolic dysfunction as measured by LV and RV peak early diastolic tissue velocity (e’) and LV Doppler early/late diastolic mitral inflow velocity ratio (E/A), among other parameters. For all parameters except LVEF, deficits were greater in very and extremely preterm than in moderate‐to‐late preterm neonates.

### 3.2 The preterm heart from childhood until adulthood

The distinct alterations of the preterm heart are not limited to the first months of extrauterine life. The Swedish EXPRESS study, investigating a cohort of 6‐year‐old children born between 22 and 26 weeks of gestation using echocardiography, showed that these children exhibited significantly smaller left ventricles with a lower LV mass adjusted to body size. Their left ventricles also exhibited functional deficits, with a more concentric contraction and a diastolic filling pattern consistent with a stiffer wall (Mohlkert et al., 2018). The same cohort demonstrated deviations in the right heart as well, as they had smaller right atria, right ventricles with smaller widths and a higher relative wall thickness, as well as higher estimated pulmonary vascular resistance (Mohlkert et al., 2020).

The first detailed cardiac study in adults born preterm was done using CMR and the creation of a unique computation cardiac atlas, which showed that young adults born preterm have a unique cardiac phenotype (Lewandowski, Augustine, et al., 2013). Preterm‐born adults were found to have shorter left ventricles with reduced volumes and an apical displacement. Furthermore, prematurity was associated with a reduction in LV longitudinal systolic, diastolic, and rotational function. The right ventricle was also altered in adults born preterm (Lewandowski, Bradlow, et al., 2013), including smaller right ventricles, as well as a significantly lower right ventricular ejection fraction (RVEF). These relative differences compared to their term‐born peers in structure and function appear to be greater in the right than in the left ventricle (Lewandowski, Bradlow, et al., 2013; Mohamed et al., 2020).

**FIGURE 1:**
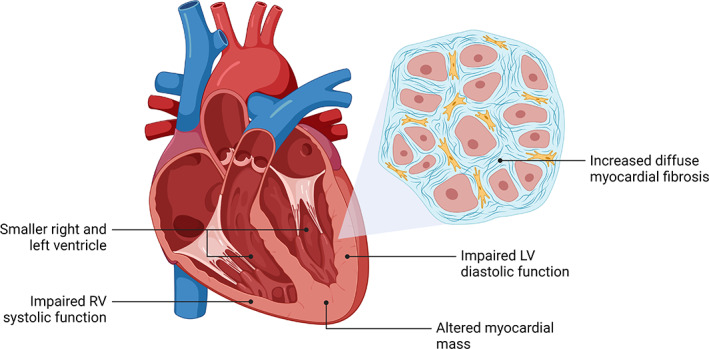
Altered cardiac structure and function in adults born preterm. Preterm birth has been associated with distinct structural and functional cardiac properties across different developmental stages, including adulthood. LV, left ventricular; RV, right ventricular

The effect of prematurity on structural and functional cardiac parameters from birth until young adulthood was summarized by a recent meta‐analysis (Telles et al., 2020). Overall, the cardiac effects of premature birth manifest themselves across the spectrum of developmental stages and include smaller ventricular internal dimensions and impaired systolic and diastolic function (Figure 1) (Flahault et al., 2021; Goss et al., 2020; Huckstep et al., 2018; Kowalski et al., 2016; Lewandowski et al., 2021; Lewandowski, Augustine, et al., 2013; Lewandowski, Bradlow, et al., 2013; Mohamed et al., 2020, 2021; Mohlkert et al., 2018, 2020; Telles et al., 2020). Adults born preterm have since been shown to have greater myocardial diffuse fibrosis, characterized by excessive extracellular volume fraction by CMR, compared to their term‐born counterparts (Lewandowski et al., 2021). Additionally, in this study it was shown that lower gestational age, as well as E/A ratio and longitudinal diastolic strain rate, were associated with increased diffuse myocardial fibrosis, suggesting the latter to be a mediator of the diastolic dysfunction seen in the preterm‐born adults. Furthermore, no associations between greater diffuse myocardial fibrosis and higher LV mass or wall thickness were found. This combination of findings supports the hypothesis that the higher myocardial mass in the first postnatal months results from cardiomyocyte hypertrophic growth, which may be caused by the immature myocardium's high susceptibility to the hemodynamic changes during the fetal‐to‐neonatal transition (Finnemore & Groves, 2015; Tan & Lewandowski, 2020). Altogether, this human study validates the findings of myocardial interstitial fibrosis and collagen deposition in animal models of prematurity (Bensley et al., 2010; Bertagnolli et al., 2014) and provides a potential mechanistic pathway underlying the diastolic dysfunction seen in earlier cohort studies of preterm‐born individuals at different ages of life (Aye et al., 2017; Lewandowski, Augustine, et al., 2013; Lewandowski, Bradlow, et al., 2013; Mohlkert et al., 2018; Telles et al., 2020).

### 3.3 The cardiac phenotype of prematurity is heterogeneous

Although preterm‐born adults are consistently found to have reduced cardiac chamber sizes, there are inconsistencies in the literature concerning myocardial mass. Different studies have demonstrated that preterm‐born adolescents and adults display a hypertrophic heart, with greater LV and RV masses (Huckstep et al., 2018; Lewandowski, Augustine, et al., 2013; Lewandowski, Bradlow, et al., 2013; Mohamed et al., 2020; Telles et al., 2020), yet studies describing other preterm‐born cohorts with lower masses also exist (Goss et al., 2020; Kowalski et al., 2016). A possible explanation for this lies within the differences between study populations. Of the studies reporting lower ventricular masses, Goss et al. (2020) investigated adolescents and young adults who were born before or at 32 weeks of gestation, while Kowalski et al. (2016) examined adolescents born before 28 weeks of gestation. The investigators of the EXPRESS study showed similarly lower LV masses in children born before 27 weeks of gestation but were unable to assess RV masses through echocardiography (Mohlkert et al., 2018, 2020). These study populations consist of people born preterm at earlier gestations than the populations displaying increased biventricular masses, meaning that they underwent an earlier disruption of their fetal growth. It is thus possible that in people that are born more prematurely, the extrauterine hypertrophic growth pattern of cardiomyocytes replaces the intrauterine hyperplastic growth pattern of cardiomyocytes at an earlier period in development, causing a greater reduction in cardiac endowment. Thus, despite the preterm cardiomyocytes still being hypertrophied, the reduction in endowment could result in an overall smaller myocardial mass, which would be the case for those born at the earliest gestations (Lewandowski & Levy, 2021).

However, the degree of prematurity is not the only factor that may affect the phenotype of the preterm heart. Various early life pathophysiological and environmental factors related to maternal and neonatal disease, as well as care around the time of delivery, have been shown to exert effects on later cardiovascular development. In addition, cardiovascular risk factors later in life may further impact the geometry and function of the preterm heart. For instance, in a recent CMR imaging study of 468 young adults, of which 200 were preterm‐born, the extent to which blood pressure elevation affected LV remodeling was explored (Mohamed et al., 2021). It was shown that the unique structure and function of the preterm left ventricle was more susceptible to systolic blood pressure elevation. Indeed, for every 1‐mmHg elevation in blood pressure, very and extremely preterm‐born adults showed a 2.5‐fold greater change in indexed LV mass compared to term‐born adults, with a 1.6‐fold greater change in indexed LV mass in moderately preterm‐born adults compared to term‐born adults. Furthermore, LV mass to end‐diastolic volume ratio per 1‐mmHg systolic blood pressure elevation in the very and extremely preterm‐born young adults was 3.4‐fold greater compared with those born moderately preterm and 3.3‐fold greater compared with those born at term. The increased prevalence of hypertension (Crump et al., 2011, 2020) and the higher blood pressures (de Jong et al., 2012) in preterm‐born adults may therefore further accelerate disease progression due to the greater susceptibility of the preterm heart to elevated blood pressures. Blood pressure management in this population may therefore be even more critical.

### 3.4 Impaired cardiac response to physiologic stress

Preterm‐born individuals have a lower peak VO_2_ (Edwards et al., 2015; Huckstep et al., 2021) and an impaired heart rate recovery (Haraldsdottir et al., 2018, 2019; Huckstep et al., 2021; Karvonen et al., 2019). Furthermore, autonomic function in preterm‐born individuals is disrupted and likely plays a role in the reduced post‐exercise recovery (Patural et al., 2004; Yiallourou et al., 2013). Pulmonary physiology, however, does not explain the reduced aerobic exercise capacity in the average preterm‐born adult (Huckstep et al., 2021). Rather than respiratory deficits, the preterm heart seems to play a critical role in this impaired exercise capacity. Indeed, the LV response to physiological stress is impaired in preterm‐born young adults, as characterized by a lower EF and cardiac output during mid‐ to high‐intensity exercise (Haraldsdottir et al., 2020; Huckstep et al., 2018). Interestingly, as reported by Barton et al. (2021) in a cohort of prematurely born adults with normal baseline function, exposure to hypoxic environment resulted in an exaggerated RV contractile response. Although this indicates possible RV contractile myocardial reserve, volumetric reserve is limited (Goss et al., 2018; Huckstep et al., 2018).

## 4 THE ROLE OF MATERNAL AND EARLY LIFE ENVIRONMENT

**FIGURE 2:**
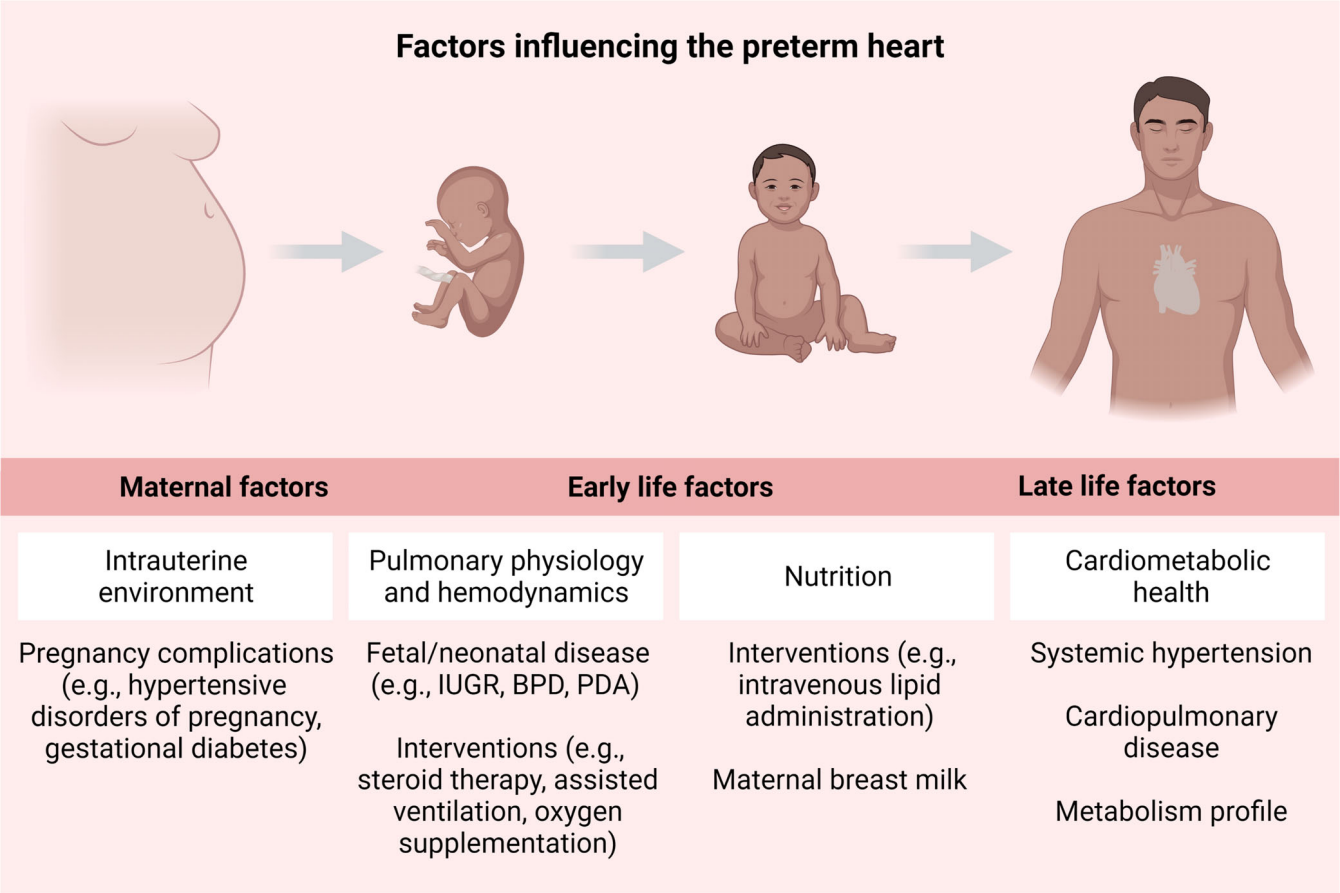
Factors influencing the preterm heart. A growing body of evidence highlights potential—beneficial or detrimental—impacts of various prenatal and postnatal factors on the cardiac phenotype of prematurity. BPD, bronchopulmonary dysplasia; IUGR, intrauterine growth restriction; PDA, patent ductus arteriosus

Prematurity is associated with a variety of factors potentially contributing to cardiac alterations (Bensley et al., 2016). These include maternal factors, associated complications of prematurity, clinical interventions, and nutritional factors (Figure 2). In the following paragraphs, examples of each of the aforementioned categories and their potential contributions to the preterm heart will be briefly discussed.

### 4.1 Maternal factors

Maternal risk factors for delivering preterm include previous preterm delivery, black race, low maternal body‐mass index, infection, and other maternal medical disorders, including hypertensive disorders of pregnancy (Goldenberg et al., 2008). Hypertensive disorders of pregnancy, and in particular preeclampsia, appear to have a unique impact on the long‐term vascular and cardiac physiology in the preterm infant (Frost et al., 2021). Preeclampsia complicates 3–5% of pregnancies globally and is an antecedent to between 15 and 30% of preterm births (Mol et al., 2016). It is a complex multisystem disorder and ranges from mild hypertension with proteinuria to eclampsia with severe hypertension, proteinuria, and multiorgan involvement (Roberts et al., 2013). A rise in systemic vascular resistance, endothelial dysfunction, and platelet activation affect the uteroplacental circulation and create a hypoxic intrauterine environment (Hutter et al., 2010; National High Blood Pressure Education Program Working Group, 2000; Redman, 1990). The fetus can adapt to this reduced oxygen supply by redistributing its blood to its vital organs, creating a hemodynamically altered bloodstream (Rizzo et al., 1992; Sun et al., 2020). Despite this, offspring of preeclamptic pregnancies may be put at unique risk for cardiac remodeling and long‐term cardiovascular disease (Andraweera & Lassi, 2019; Kajantie et al., 2009; Timpka et al., 2016). Preeclampsia or gestational hypertension resulting in preterm birth has been shown to associate with a threefold greater risk of being hypertensive by age 20 years (Davis et al., 2015). Further to this, in infancy, hypertension during pregnancy was shown to predict an increase in RV mass during the first three postnatal months, independent of preterm birth (Aye et al., 2017). Preterm‐born adults born to preeclamptic pregnancies have also been shown to have an overall reduction in LV longitudinal peak systolic strain compared to their preterm‐born peers born to normotensive pregnancies (Lewandowski, Augustine, et al., 2013). Although hypertensive pregnancies have been linked to cardiac consequences after both term and preterm birth in the offspring, further research is needed to understand the role of these and other maternal factors in long‐term cardiac remodeling in the offspring.

### 4.2 Associated complications of prematurity

Prematurity is not only associated with maternal conditions but also with fetal and neonatal complications that may exert stress on the immature cardiovascular system. For instance, intrauterine growth restriction (IUGR) is a fetal condition that occurs in more than 20% of preterm births (Gardosi, 2005). It is the pathological counterpart of small‐for‐gestational‐age, reflecting poor fetal growth during pregnancy through placental failure of delivering an adequate supply of oxygen and nutrients (Zohdi et al., 2012). At least three different fetal cardiac phenotypes are reported to be induced by IUGR: an elongated phenotype (which should not necessarily be interpreted as abnormal), a globular phenotype in late‐onset IUGR, and a hypertrophic phenotype occurring in severe and early IUGR (Rodriguez‐Lopez et al., 2017; Van Mieghem, 2017). Additionally, children born IUGR have been shown to have smaller hearts with non‐hypertrophic spherical ventricles and impaired relaxation, all independent of body size (Sarvari et al., 2017). Although preterm‐born children with IUGR have been shown to have the greatest cardiac remodeling (Sarvari et al., 2017), alterations have also been observed by echocardiography and CMR imaging in mild IUGR cases born at term (Crispi et al., 2010, 2021) suggesting a distinct contribution of IUGR on the cardiac phenotype of prematurity.

An additional complication and the most common morbidity of prematurity is bronchopulmonary dysplasia (BPD), although incidences vary between regions and institutions (Davidson & Berkelhamer, 2017; Thebaud et al., 2019). An important complication of impaired bronchoalveolar development is the often associated pulmonary hypertension (PH), which leads to 2‐year mortality in more than half of preterm infants when persevering beyond 2 months after birth (Khemani et al., 2007). PH might develop from BPD due to factors involving inflammation and endothelial dysfunction maintained by alveolar hypoxia and is characterized by an elevated pulmonary vascular resistance and resultant RV afterload (Bates et al., 2020; Hansmann et al., 2021; Lignelli et al., 2019; Thebaud et al., 2019). Consequently, this translates into abnormalities of cardiac function and structure. Although no conventional echocardiographic measures of LV function have been shown to be altered based on BPD severity or presence of PH, asynchronous movement of the ventricular walls appears to be significantly prolonged in neonates with severe BPD‐PH (Torres et al., 2021). The right ventricle has been shown to be more affected by BPD‐PH, and long‐term subclinical deficits in RV function have been reported accordingly in those previously diagnosed with BPD‐PH (Blanca et al., 2019; Kwon et al., 2016). Even though a small cohort of prematurely born adults showed impaired RV‐vascular coupling (Mulchrone et al., 2020), the extent of the pulmonary physiology contribution to changes in the preterm heart remains uncertain. For example, RV contractile reserve in response to hypoxia has been shown to remain unaffected by neonatal history of BPD in very and extremely preterm‐born adults (Barton et al., 2021), and functional RV deficits to be independent of pulmonary physiology in a cohort of moderately preterm‐born adults (Mohamed et al., 2020). On the other hand, a recent study by Dartora et al. (2021) showed that young adults born before 30 weeks of gestation showed increased RV dysfunction when they had a history of BPD. These findings emphasize the need for further longitudinal research in individuals born preterm exploring the effects of BPD, chronic lung disease, and PH on long‐term cardiac structure and function.

### 4.3 Clinical interventions

Despite premature birth being associated with high levels of neonatal morbidity, survival rates of even the most premature newborns have drastically risen during the last decades, with contemporary survival rates of nearly 90% in neonates born between 22 and 32 weeks of gestation when admitted to neonatal intensive care units (Hack & Fanaroff, 2000; Larroque et al., 2004). This shift in survival is most often ascribed to the combined effects of assisted ventilation, surfactant therapy, and antenatal steroid therapy; some of the greatest successes in neonatal medicine, which were established in the 1980s (Hack & Fanaroff, 2000). With these advancements, however, new exposures that may have detrimental effects on long‐term cardiac structure and function have emerged.

Perinatal corticosteroid therapy has been linked with adverse cardiac outcomes in preterm‐born individuals (Vrselja et al., 2021). Antenatal corticosteroids are routinely administered when there is a risk of preterm birth to accelerate fetal lung maturation, while postnatal corticosteroids are given to preterm newborns as a treatment for BPD (Purdy, 2004; Purdy & Wiley, 2004). Antenatal administration, in particular, reduces the risk of neonatal mortality and respiratory morbidity, providing the greatest benefit to neonates born extremely preterm (McGoldrick et al., 2020; Travers et al., 2017). The use of low‐dose postnatal corticosteroids is associated with improved respiratory outcomes and is recommended for neonates at the highest risk of respiratory complications (Doyle, 2021; Sweet et al., 2019). Most current recommendations, however, advise against the clinical implementation of high‐dose postnatal corticosteroids due to associations with increased risk of developing cerebral palsy (Jefferies, 2012). In addition, multiple studies have revealed associations between both antenatal and postnatal corticosteroids and cardiac irregularities. Antenatal and postnatal corticosteroid exposure, for instance, have been associated with a transient LV hypertrophy in infancy (Choudhry et al., 2018; Cox et al., 2019; Skelton et al., 1998). In spite of this, data on long‐term structural and functional cardiac effects associated with corticosteroid exposure are less clear. In children previously exposed to postnatal corticosteroids, resting hemodynamic data were not significantly different (de Vries et al., 2008), although they showed an impaired cardiovascular stress response (Karemaker et al., 2008). Furthermore, in utero corticosteroid exposure has been linked to cardiometabolic effects reaching into early adulthood, as maternal exposure to antenatal corticosteroids showed to be associated with elevated ascending aorta and aortic arch stiffness, as well as altered glucose metabolism in preterm‐born offspring (Kelly et al., 2012). Future information from large prospective investigations and mechanistic animal research are likely to give us more insight into the relationship between corticosteroid exposure and its cardiovascular repercussions.

Caffeine administration, ventilation strategies, surfactant therapy, and vitamin A supplementation are also routinely applied in clinical practice to minimize lung injury (Thebaud et al., 2019). Caffeine triggers an increase in cardiac index, stroke volume, and heart rate through a pressor effect (Abdel‐Hady et al., 2015; Walther et al., 1990), while surfactant causes a dose‐dependent decrease in blood pressure through vasodilatation (Hentschel et al., 2020). Both have an immediate impact on neonatal hemodynamics, counteracting negative consequences of a patent ductus arteriosus, but long‐term cardiovascular risks seem to be limited. On the other hand, mechanical ventilation has been associated with several complications, including airway and tracheal injury, air‐leak syndromes, volutrauma, and neurologic injury (Abubakar, 2012). Preterm newborns who underwent invasive ventilation on day one after birth demonstrated impaired LV diastolic function, suggesting a substantial role of LV diastolic function in the evolution of respiratory morbidity and altered cardiopulmonary physiology (Bussmann et al., 2018). Furthermore, variations in RV mass but not function in preterm‐born adults were associated with the need for mechanical ventilation (Goss et al., 2020; Lewandowski, Bradlow, et al., 2013). Even if this variation could be influenced by underlying respiratory dysfunction, it is plausible that RV preload and afterload are also affected by changes in intrathoracic pressure and lung volume caused by mechanical ventilation (Bogaard et al., 2009; Lewandowski, Bradlow, et al., 2013; Marini et al., 1981).

Mechanical ventilation and apnea of prematurity, caused by immature respiratory control in preterm neonates, sometimes result in intermittent hypoxic events (Eichenwald et al., 2016; Martin et al., 2011). Oxygen supplementation is an established intervention to counter these periods of hypoxia but increases the risk of exposure to a hyperoxic environment (Ali et al., 2021; Oei & Vento, 2019). Various animal experimental studies, some of which include models of preterm birth, have shown a link between neonatal hyperoxia exposure and changes in cardiac structure and function, resulting in adult heart failure (Bertagnolli et al., 2014; Velten et al., 2011, 2014). In humans, neonatal hyperoxia exposure is linked to the development of BPD through the generation of reactive oxygen species triggering an inflammatory response (Jobe et al., 2008; Kalikkot Thekkeveedu et al., 2017). However, the long‐term cardiac sequelae of hyperoxia in human preterm neonates remain largely unexplored.

### 4.4 Nutritional factors

Preterm‐born neonates are often unable to maintain similar intrauterine growth rates after birth, resulting in a higher need for nutrients during the neonatal period (Hamayun et al., 2021; Hay Jr., 2018; Horbar et al., 2015). This causes undernutrition and nutritional imbalances such as hyperglycemia to be common in preterm newborns admitted to the neonatal intensive care unit, suggesting that nutritional factors play a key role in their development (Hamayun et al., 2021; Hay Jr., 2018; Zamir et al., 2018). Different studies support this notion. For example, in a cohort of prematurely born individuals followed up since birth, neonatal intravenous lipid exposure caused a subsequent rise in cholesterol, which was associated with aberrant aortic and myocardial function in adulthood (Lewandowski et al., 2011). Furthermore, in a cohort of extremely preterm‐born children born at <27 weeks of gestation, differences in lipid and protein intake during the first four postnatal weeks were linked to altered LV outflow tract dimensions at 6.5 years after birth (Hamayun et al., 2021). In addition to this, children who were exposed to a significant period of neonatal hyperglycemia demonstrated increased LV wall thickness, as well as higher blood pressures (Hamayun et al., 2021; Zamir et al., 2019).

The potential benefits of human milk for cardiovascular development of the preterm infant have also been studied (El‐Khuffash et al., 2020). Preterm infants with higher maternal milk consumption have recently been shown to have improved LV and RV performance (El‐Khuffash et al., 2021). This effect appeared at 36 weeks post‐menstrual age and became more significant by 1 year of age. Furthermore, the potential beneficial effect of maternal breast milk in early postnatal life may extend into adulthood. In a study of preterm‐born adults randomized to different feeding regimes at birth, those fed exclusively human milk as infants had increased LV and RV indexed end‐diastolic volumes and stroke volumes in comparison to preterm‐born individuals who were exclusively formula‐fed (Lewandowski et al., 2016).

## 5 CONCLUSIONS AND FUTURE DIRECTIONS FOR RESEARCH

Advances in neonatal care have allowed for the vast majority of prematurely born newborns to reach adulthood. It is becoming increasingly clear that preterm birth, across gestations including those born moderate‐to‐late preterm, leads to immediate and long‐term changes in cardiac structure and function (Figure 1). Additionally, a growing body of evidence links these cardiac irregularities to functional repercussions, which may play a role in long‐term cardiovascular morbidity and mortality. Important knowledge gaps still remain regarding the morphological and functional characteristics of the preterm heart and the factors influencing the cardiac phenotype of prematurity. Of note, large‐scale cohort studies with lifelong follow‐up are needed to corroborate previous findings and to determine to what extent the cardiac phenotype of prematurity exerts an effect on long‐term cardiovascular mortality. In addition to human studies, further experimental animal research will be valuable for obtaining mechanistic insight into the pathogenesis of cardiac changes seen in preterm‐born people. Further research is needed to design targeted biomarkers, prevention strategies, and intervention approaches for this population to decrease long‐term cardiovascular risk. Once more evidence becomes available in these domains, changes in clinical care might prove beneficial to the significant preterm‐born proportion of the population.

## AUTHOR CONTRIBUTIONS

**Art Schuermans:** Conceptualization (equal); writing – original draft (lead); writing – review and editing (equal). **Adam Lewandowski:** Conceptualization (equal); supervision (lead); writing – original draft (supporting); writing – review and editing (equal).
